# Phenotypical Variation of Ruminal Volatile Fatty Acids and pH during the Peri-Weaning Period in Holstein Calves and Factors Affecting Them

**DOI:** 10.3390/ani12070894

**Published:** 2022-03-31

**Authors:** Panagiota Kazana, Nektarios Siachos, Nikolaos Panousis, Anatoli Petridou, Vasilis Mougios, Georgios E. Valergakis

**Affiliations:** 1 Laboratory of Animal Husbandry, Faculty of Veterinary Medicine, School of Health Sciences, Aristotle University of Thessaloniki, P.O. Box. 393, 54124 Thessaloniki, Greece; pkazana@vet.auth.gr (P.K.); nsiachos@vet.auth.gr (N.S.); 2 Clinic of Farm Animals, Faculty of Veterinary Medicine, School of Health Sciences, Aristotle University of Thessaloniki, 54124 Thessaloniki, Greece; panousis@vet.auth.gr; 3 Laboratory of Evaluation of Human Biological Performance, School of Physical Education and Sport Science, Aristotle University of Thessaloniki, 54124 Thessaloniki, Greece; apet@phed.auth.gr (A.P.); mougios@phed.auth.gr (V.M.)

**Keywords:** rumen volatile fatty acids, dairy calf, weaning, management factors

## Abstract

**Simple Summary:**

The evolutionary course of rumen volatile fatty acids and their association with management factors during the peri-weaning period under field conditions have not been sufficiently investigated. There is a large phenotypical variability among calves in rumen parameters. There are several management factors affecting them, associated with a smooth rumen adaptation peri-weaning. The results enhance our knowledge about management practices that optimize ruminal adaptation during the weaning process.

**Abstract:**

Two hundred and forty-three clinically healthy Holstein calves from eight commercial dairy farms were used to: (a) describe the evolutionary course of ruminal VFA concentration and pH during the peri-weaning period and (b) assess management factors affecting their phenotypical variation of these parameters. Management practices were recorded individually for each calf as these were not fixed within farms. Samples of ruminal fluid were collected at −7 d, 0 d, and 7 d relative to weaning. Gas chromatography was used to measure ruminal VFAs, and pH was measured on site. Linear mixed models for repeated measurements were used to assess the effects of management factors and their interactions. A large among-calves phenotypical variability was observed. Estimated marginal means showed that concentrations of acetate, butyrate, and total VFAs (but not of propionate) significantly decreased, while acetate propionate increased, from −7 d to 7 d. Age at weaning and body weight at −7 d were positively associated with total and several individual VFA concentrations. Group housing and late forage feeding pre-weaning were associated with higher VFA concentrations; the same factors, as well as step-weaning, were associated with pH values around 6. Feeding 7–8 L of milk replacer daily did not preclude a smooth transition, irrespective of weaning method.

## 1. Introduction

Calves are born with a nonfunctional and underdeveloped rumen and initially depend on milk to meet their nutrient requirements [1]. Globally, most dairy calves are immediately separated from their dams and artificially reared for at least 5–6 weeks [1,2]. During this period, the aim is to accelerate anatomical and metabolic changes of the digestive tract, gradually switching nutrient supply from liquid to solid feed. Weaning, the complete substitution of concentrates and forages for milk or milk replacer (MR), is a crucial transition period; adaptation failure is associated with increased disease risk and reduced growth or even weight loss [3].

Early intake of solid feed promotes microbial proliferation and volatile fatty acid (VFA) production, thus stimulating ruminal epithelium development [4]. Acetate (C2), propionate (C3), and butyrate (C4) are the main ruminal VFAs formed by the fermentation of carbohydrates. Type and chemical composition of solid feed affects ruminal environment and VFA production. Acetate is an end product of fiber fermentation—large amounts of C2 are negatively associated with size and development of ruminal papillae and positively associated with ruminal volume [5]. Butyrate, a fermentation end product of sugars and fiber, greatly influences ruminal papillae development, while being the main energy source of epithelial cells [5]. Propionate, resulting mainly from starch fermentation, plays a similar role to C4, albeit secondary, regarding ruminal development.

Less is known regarding minor VFAs in calf rumen fluid. Valerate (C5) is produced by lactate-utilizing bacteria, indicating lactate accumulation in the rumen [6]. Its concentration in adult cows has been associated with subacute ruminal acidosis [7,8,9]. No such association has been reported in weaning-age calves yet. Isovalerate (iso-C5) and isobutyrate (iso-C4) are branched-chain fatty acids originating from the deamination of several amino acids in the bacterial cell. Both are considered growth factors for cellulolytic bacteria. Consequently, as cellulolytic activity increases across weaning due to higher forage intake, iso-C5 and iso-C4 concentration is considered to decline [6]. Carbohydrate fermentation and VFA production are reflected in ruminal fluid pH. Peri-weaning, calves usually have lower pH values than adult cows, mostly due to higher dietary inclusion of concentrates and lower buffering capacity [10]. However, Laarman and Oba [11] found that daily average pH values of calf ruminal fluid were similar to those of adult cows, noting that values below 5.8 indicate rumen acidosis in calves as well. Acetate-to-propionate ratio, an indicator of acidosis risk in adult cows, has not been considered for calves. Preliminary analysis of our data suggested that different pH evolution patterns exist around a pivotal pH value of 6.0 [12]. Elevated VFA formation and a healthy ruminal environment, as indicated by ruminal fluid pH, depict a smooth adaptation of the rumen during the peri-weaning period.

Several management strategies (age and method of weaning, feeding, and housing practices) have been reviewed [1,2] and are supposed to affect the fermentation profile, but there are no established ranges of VFA concentrations, C2, C3, and pH, that are considered as optimum; moreover, these strategies have been generally evaluated independently from one another [1,13,14].

Studies with repetitive sampling of calf rumen contents in order to measure VFA concentrations and pH are not common and are not focused on describing the evolutionary progression of VFA and pH during the peri-weaning period. Instead, time intervals between sampling are long and most of them are trials, aiming to compare the effect of different management practices one at a time: dietary treatments [15,16], feed processing [4,17], and weaning strategies [15]. Moreover, in most cases, calves were reared on the same farm under controlled experimental conditions and the effect of weaning has not been assessed using repeated measurement analysis. The phenotypical variation of VFA concentrations and pH has not been described; However, this is important in an effort to optimize ruminal adaptation during the weaning process.

Therefore, the objectives of this study were: (a) to describe the evolutionary course of VFA concentration and pH during the peri-weaning period in Holstein calves under field conditions, and (b) to assess management factors affecting phenotypical variation of ruminal fluid VFAs and pH.

## 2. Materials and Methods

The study was conducted with the approval of the Research Committee of Aristotle University of Thessaloniki, Greece (Protocol number 576/30.10.2015) and in accordance with the institutional guidelines. The farmers gave informed consent for testing procedures and including their animals in the study.

### 2.1. Farms, Animals, and Study Design

A convenience sample of eight dairy farms located in the prefecture of Central Macedonia, Greece, representative of the average Greek dairy farm, were selected. Farms were within a radius of 70 km from the city of Thessaloniki and at an altitude ranging from 50 to 200 m. Farms kept between 110 and 360 milking Holstein cows, and milk yield ranged from 9000 to 12,000 kg per cow per lactation.

This observational cohort study was conducted from April 2017 to November 2019. Two-hundred and forty-nine purebred Holstein pre-weaned calves were initially enrolled. Only clinically healthy calves, as assessed by clinical examination and without any history of diseases from birth until the start of our study, were included. Inclusion criteria were alertness, normal appetite, rectal temperature (>38 and <39.5 °C), and absence of dehydration, diarrhea, cough, and nasal or ocular discharge [18].

### 2.2. Recording of Management Practices

All calves were fed 3–4 L of colostrum within 12 h from birth; 2 to 4 days later, all calves were transitioned to MR feeding. During the pre-weaning period, MR was prepared according to manufacturers’ guidelines (12.5% solids on all farms) and offered in volumes ranging from 4 to 8 L daily, in simple buckets at 39 °C, divided in 2 equal meals. Starter in meal form (ground) was introduced during the 1st week of age. When provided, forage was either alfalfa hay or a mixture of alfalfa hay and wheat straw, offered in long form in both cases. All calves had continuously free access to clean water. Calves were moved from calving pens to rearing facilities within their first day of life. Thereafter, they were housed in hutches, elevated stalls, or straw-bedded pens, individually or in groups.

Although each farm aimed to apply a fixed rearing program, in several cases, management practices within the same farm regarding forage feeding, housing, and weaning method, were not constant due to space limitations, barn configuration, and presence of different housing facilities. For instance, weaning was supposed to be performed gradually on all farms; however, several calves were weaned abruptly to allow entry of newborns in the rearing barn.

Postweaning, calves were offered starter and clean water ad libitum, with or without forage, and kept in preweaning type of facilities individually or in groups, either switching type or not.

All previous data were recorded individually for each calf. Farm-specific management details are presented in Table 1. Overall, calves were weaned at a mean (±SD) age of 71.2 (±15.1) days and with a mean bodyweight of 72.2 (±16.5) kg, as calculated from heart girth measurements [19].

### 2.3. Samples Collection and Analysis

Ruminal fluid samples were collected from all calves at 3 time-points relative to weaning: 7 days pre-weaning (−7 d), at weaning (0 d), and 7 days post-weaning (7 d). Two people restrained each calf with minimal force and samples were collected via an oro-ruminal probe attached to a manual suction pump, 1–2 h post-feeding using the technique described by Klopp et al. [20]. Ruminal fluid pH was measured on-site with a portable device (Hanna^®^ Instruments, Woonsocket, RI, USA). Samples were placed in a portable cooler immediately after collection and were transported to the laboratory within 1–2 h, where they were placed in 1.5-mL polyethylene (Eppendorf) tubes and stored at −20 °C pending VFA analysis.

All ruminal fluid samples were analyzed to assess the concentration of C2, C3, C4, iso-C4, C5, iso-C5, caproic acid (C6), and enanthic acid (C7), using gas chromatography. Specifically, in an Eppendorf vial, (a) 10 μL of 1.083 mol/L crotonic acid as internal standard, (b) 1 g of thawed sample, and (c) 30 μL of 20% (*v*/*v*) H_2_SO_4_ were placed. Then, the vials were centrifuged at 3000× *g* at 4 °C for 10 min. After centrifugation, 1 μL of the supernatant was analyzed in an Agilent 7890A gas chromatograph (Waldbronn, Germany) equipped with ultra-inert inlet liner, a DB-WAX UI column (length 30 m, internal diameter 0.25 mm, film thickness 0.25 μm), and a flame ionization detector. The column temperature was programmed from 80 °C, held for 1 min, to 150 °C at 10 °C min^−1^. The carrier gas was helium at a constant head pressure of 24 psi, and the split ratio was 1:50. Individual VFAs were detected in the chromatograms by comparing their retention times with those of standards included in the Supelco Volatile Free Acid Mix (Bellefonte, PA, USA) and were quantified by comparing their peak areas with that of crotonic acid, after adjusting for their response factors, using the Agilent ChemStation, version B.04.03, software [21,22]. Coefficients of variation for both intra-assay precision and inter-assay variability were 0.2–1.0% and 1.5–4.9%, respectively.

### 2.4. Statistical Analysis

A total of 243 calves had complete records and their data were included in the statistical analysis. Linear mixed models for repeated measurements (LMMs) were used to assess the potential effect of the following factors on the phenotypical variation of individual and total VFA concentrations, C2:C3 ratio, and on ruminal fluid pH:

Time-point (TIME, 3 levels: −7 d, 0 d, and 7 d),
Gender (GEND, 2 levels: female and male),
Age at weaning (AGEW, covariate in continuous scale),
Body weight at −7 d (BW-7, covariate in continuous scale),
Daily volume of MR (VOLM, 3 levels: “low” (4–5 L), “medium” (6 L), and “high” (7–8 L)),
Forage administration pre-weaning (FPRE, 3 levels: “no”, “early”, (before 1st month of age) and “late” administration (after 1st month of age)),
Housing pre-weaning (HPRE, 2 levels: individual and group housing),
Method of weaning (METW, 2 levels: abrupt and step),
Switch housing at weaning (HSWI, binary),
Forage inclusion post-weaning (FPOS, binary),
Housing post-weaning (HPOS, 2 levels: individual and group).

The last four factors (METW, FPOS, HSWI, and HPOS) begin to affect the variation of the dependent variables after −7 d. Therefore, they were all assigned the same coding at −7 d; METW was assigned different coding at 0 d and 7 d, and HSWI, FPOS, and HPOS were assigned different coding at 7 d only.

Time-points were used to specify within-subjects repeated observation. The random effects of herd and calf were considered by building a nested term (each calf nested within each herd). Among diagonal, compound symmetry and first-order autoregressive matrix, the appropriate covariance structure for random effects was selected, based on the lower Akaike’s information criterion value. All factors mentioned above, as main effects and all two-way interactions, were assessed as independent variables in the LMMs. Final LMMs were built by manual backward elimination of variables, with a non-significant effect set at the 0.10 level.

Assumptions of normality and homoscedasticity were assessed by visually observing the Q-Q plots and the predicted values vs. residual plots, respectively. Multicollinearity issue was precluded by performing several diagnostic tests for regression models (correlation matrix, variance inflation factor, condition index at the lowest eigenvalue row, and variance proportion). At significant F values for factors with >2 levels, pairwise comparisons between the estimated marginal means for each level were performed using the Bonferroni confidence interval adjustment. Regression coefficients produced for covariates were used to quantify their contribution to dependent variables.

## 3. Results

Descriptive data regarding ruminal VFA concentrations at the three selected time-points (−7 d, 0 d, and 7 d) and during the whole peri-weaning period (“overall”) are presented in Table 2. A rather large variability was observed, coefficients of variation being 30%, 54%, 45%, for the overall C2, C3, and C4 concentrations, respectively, and even larger for the other VFAs. The variability of overall total VFA concentration was 35%. On the other hand, mean C2:C3 ratio slightly increased from −7 d to 7 d, but it remained <2 (Table 2). The 10th and 90th percentiles of pH were 5.2–7.0 (median: 6.10), 5.3–7.2 (median: 6.48), and 5.2–7.1 (median: 6.30), at −7 d, 0 d, and 7 d, respectively.

Estimated marginal means (EMM) show the variation of volatile fatty acid concentration and of pH, where there is a significant effect from time-points, and are presented in Table 3.

All explanatory variables are included as main effects in LMMs, which had a significant effect on ruminal VFA concentrations and on ruminal fluid pH as shown in Table 4. Their estimated marginal means (EMM) are shown in Table 5.

Total VFA concentration decreased by 7.8%, from −7 d to 7 d (Table 3, *p* = 0.028). Both AGEW and BW-7 were positively associated with total VFA concentration (Table 4); for each 1-day and 1-kg increase, total VFA concentration increased by 0.43 mmoL/L (*p* = 0.028) and 0.28 mmoL/L (*p* = 0.003), respectively. Additionally, “late” forage feeding resulted in higher total VFA concentration, compared to “no” and “early” forage feeding, by 15.2% (*p* = 0.003) and 16.6% (*p* < 0.001), respectively. Moreover, group housing pre-weaning was associated with increased total VFA concentration, by 15.6%, (*p* < 0.001) (Table 5).

Acetate concentration decreased by 7.2%, from −7 d to 7 d (Table 3, *p* = 0.013). A 1-kg increase of BW-7 was associated with a 0.13 mmoL/L increase of C2 (*p* = 0.001). Moreover, C2 concentration decreased by 0.15 mmoL/L for each 1-day of AGEW increase (*p* = 0.019). Group housing pre-weaning was positively associated with C2 concentration by 13.1% (Table 5, *p* < 0.001), whereas individual housing was not. No association of TIME with C3 concentration was detected (Table 3), while a 1-kg of BW-7 increase resulted in an increase of C3 concentration by 0.17 mmoL/L (*p* = 0.017). Late forage feeding resulted in higher C3 concentration, compared to both “no” and “early” feeding, by 32.3% and 34.6%, respectively (*p* < 0.001). Additionally, C3 concentration was positively associated with group HPRE (*p* < 0.001) but negatively with group HPOS (*p* = 0.034). “Early” forage feeding was associated with a higher C2:C3 ratio compared to “late” feeding (*p* < 0.001). Individually housed calves had a significantly higher ratio pre-weaning (*p* < 0.001) but a lower post-weaning one (*p* < 0.001) compared to group-housed calves (Table 5).

Butyrate concentration decreased by 34.73% from −7 d to 7 d (Table 3, *p* = 0.010). Feeding no forage post-weaning resulted in lower C4 concentrations (*p* = 0.012). Isobutyrate concentration decreased from −7 d to 7 d by 16.6% (*p* = 0.029). Switching housing at weaning negatively affected iso-C4 concentration by 45.9% (*p* = 0.006). Individual pre-weaning housing was positively associated with iso-C4 concentration by 33.0% (*p* = 0.004). On the contrary, individual post-weaning housing calves had lower iso-C4 concentration by 47.9% (*p* = 0.016) (Table 5).

Valerate concentration decreased from −7 d to 0 d by 12.2% (Table 3, *p* = 0.004). Both AGEW and BW-7 were positively associated with C5 concentration (Table 4); for each 1-day and 1-kg increase, C5 concentration increased by 0.024 mmoL/L (*p* = 0.022) and 0.021 mmoL/L (*p* = 0.007), respectively. Feeding a “high” amount of MR resulted in lower C5 concentration, compared to feeding a “low” or “medium” amount of MR, by 46.4% (*p* < 0.001) and 27.9% (*p* = 0.033), respectively. Moreover, “late” forage feeding resulted in higher C5 concentration, compared to “no” and “late” feeding, by 54.0% and 36.3%, respectively (*p* < 0.001). Additionally, group housing post-weaning was negatively associated with C5 concentration (*p* = 0.004). Iso-valerate concentration decreased from −7 d to 7 d by 26.3% (*p* = 0.001). A 1-day increase of AGEW resulted in an increase of iso-C5 concentration by 0.01 mmoL/L (*p* = 0.001). Moreover, iso-C5 concentration decreased by 0.01 mmoL/L for each 1-kg of BW-7 increase (*p* = 0.001). Additionally, group housing post-weaning was positively associated with iso-C5 concentration (*p* = 0.016) (Table 5).

Caproate concentration was positively associated with AGEW (*p* = 0.019) and negatively with “no” forage feeding (Table 4, *p* = 0.020). Enanthate concentration was positively associated with “late” forage feeding (S1, *p* = 0.017).

The ruminal pH did not differ among the three time-points (Table 3). Calves weaned abruptly had significantly higher pH values compared to those weaned gradually (6.70 vs. 6.04, *p* = 0.001). “Early” forage feeding resulted in higher pH values (*p* < 0.05) compared to “no” and “late” feeding (6.54 vs. 6.23 and 6.13, respectively, *p* < 0.05). Calves housed individually pre-weaning had significantly higher ruminal pH values by 10.7% compared to group-housed ones (*p* < 0.001) (Table 5).

Notable interactions (Table 6) included the following: (a) TIME × VOLM: in Low VOLM fed calves, C2 and C3 concentrations decreased significantly on 0 d, while on 7 d C3 significantly increased and C2:C3 and pH significantly decreased; (b) TIME × FPRE: while “late” FPRE resulted in rather steady conditions (C3 concentration, C2:C3 ratio and pH), “early” FPRE had increased C3 and decreased C2:C3 ratio on 7 d only; (c) TIME × HPRE: C2:C3 ratio and pH in group-housed calves were significantly lower on 7 d; and (d) VOLM × METW: in medium VOLM fed calves, total VFA and C3 concentrations were higher, while C2:C3 ratio and pH were lower following the step method. Details regarding variables as two-way interactions with significant effect on ruminal VFA concentrations and on ruminal fluid pH are presented as estimated marginal means in Supplementary Materials (Tables S1–S10).

## 4. Discussion

To our knowledge, this is the first large-scale multi-farm observational study under field conditions reporting ruminal VFA concentrations and pH during the peri-weaning period. In most previous studies, which, admittedly, had different objectives under controlled experimental conditions, only mean values are reported for a comparable calf weaning age; variation is not analyzed or discussed [4,15,16,23,24]. In the present study, mean VFA values appear to be similar to those reported in some, but not all, studies. Regarding pH values, similar [11,15] and higher ones [25,26,27] than ours have been reported. These diversities are probably due to differences in feeding programs and overall management practices, such as a lower weaning age. Moreover, in most cases, calves were sampled only once; therefore, the evolutionary course of VFA concentrations and pH during the peri-weaning period, which was one of the objectives of this study, has never been described before.

Decreased total VFA production through weaning was mainly due to decreased production of C4; major changes occurred after weaning day (0 d). Butyrate is the most bioactive VFA, used as fuel by the epithelial ruminal cells, and stimulates the development of ruminal papillae, a process completed at about 2–3 months of age [4]. Even if temporary, this decrease is an undesirable development. Absorbed C4 is oxidized to ketones [1,28]; β-hydroxybutyrate, the most abundant of those, is considered an indicator of ruminal epithelium maturation [29] and can be used to determine sufficient starter intake and rumen development for a successful transition across weaning [30]. The decrease in C2:C3 ratio was due to the decrease of C2 concentration, since C3 did not differ among the three time points. The combined decline of C2 and C4 concentrations around weaning indicates decreased forage consumption of both forage and starter. Weaning stress, a well described phenomenon in the literature [1], supposedly results in decreased solid feed intake, but research has not provided a distinction between forage and concentrates so far. On the other hand, the decrease in iso-C4 and iso-C5 is a favorable development.

The large among-calves variability in ruminal VFA concentrations and their changes through weaning makes their course difficult to interpret and implies that distinct evolutionary patterns exist, probably associated with specific management practices. This is an interesting subject for future research.

Successful ruminal adaptation to an exclusive solid diet is a concept easy to understand but difficult to define. Ruminal parameters are obvious candidates for such an assessment, but recommended values or ranges for ruminal VFA concentrations, C2: C3 ratio and pH for calves are not available for weaning-age calves. Established values for adult cattle cannot serve as standards, because dry matter intake (expressed as a percentage of BW), forage to concentrate ratio markedly differ between them and weaning-age calves. Higher values would be preferable—based on data presented in Table 2 and Table 3, total VFA concentrations between 100–140 mmol/L represent above average feed intake and fermentation. Moreover, stable or slightly decreasing C3 and C4 concentrations reflect a consistency in starter intake during the transition period; a similar course is desirable for C2 as well. A rather low mean C2: C3 ratio of around 1.8 and a pH range of 6.0–6.2 corresponds to the above VFA range, but variability was large.

Management factors had several interesting effects on phenotypical variation of ruminal fluid VFA and pH. “Late” FPRE resulted in higher total VFA, C3, and C5 concentrations, as well as lower C2: C3 ratio and pH values, probably reflecting a higher starter intake [1]. “Early” forage inclusion in the diet discourages concentrate intake; lower total VFA and C3 concentrations imply lower starter intake. This is not desirable at this stage, when rapid rumen development is the main goal [31]. “No” FPRE was not beneficial either; total VFA and C3 concentrations were also low, as was the case with ‘’early’’ forage inclusion. “Late” FPRE seemed to have the most favorable effect on ruminal parameters; this is corroborated by the TIME × FPRE interactions detected. It must be noted that our results apply only when forage (alfalfa or a mixture of alfalfa hay and wheat straw) is offered in long form. Feeding FPOS resulted in higher C4 concentration, a beneficial development. One issue that preoccupy researcher is whether bedding consumption affects comparisons among forage feeding systems. Hill et al. [32] showed minimum effects on bedding consumption; in the present study, wheat straw was available as bedding to all calves, and therefore should not have affected our results.

Previous studies reported that gradual weaning improves solid feed intake and ruminal fermentation [28,33]. In the present study, METW had no association with VFA concentrations; pH was higher in abruptly weaned calves, ranging from 6.4 to 6.9 and indicating reduced solid feed intake and impaired adaptation [15]. Gradual weaning appears to be the method of choice when a medium VOLM is offered.

Body weight is positively associated with dry matter intake [34]. Therefore, increased total VFA, C2, C3, and C5 concentrations in heavier calves was an anticipated finding. Calves with higher BW-7 showed a better adaptation around weaning. This strengthens feeding recommendations for high daily gains during the nursery period [35].

The positive association of AGEW with total VFA, C4, C5, and iso-C5 concentrations is in agreement with previous studies [25,33], reporting enhanced solid feed (mainly starter) intake and ruminal development following late weaning. Moreover, increased age at weaning was negatively associated with C2 concentration, probably indicating a low forage consumption. Component feeding was common in the farms that participated in our study, favoring concentrates against forage intake.

Group housing pre-weaning was associated with higher total VFA, C2, and C3 concentrations and lower pH, indicating higher solid feed intake. Social housing results in increased competition for feed resources and higher concentrate consumption [35]. Previous studies reported better performance, in terms of daily gain, during the pre-weaning period for group-housed calves [36,37,38]. To our knowledge, the association of group housing with improved ruminal adaptation during the peri-weaning period has not been described before. The higher C3 and the lower pH and C2:C3 ratio post-weaning indicate a higher concentrate consumption from calves that were group-housed pre-weaning. This was amplified post-weaning, as shown by the TIME × HPRE interaction on 7 d (low C2:C3 ratio and pH).

Group HPOS was associated with lower C3 and C5, as well as higher iso-C4, iso-C5, and C2:C3, indicating decreased feed intake. The stress associated with the immediate post-weaning group housing for the majority of individually housed calves pre-weaning could account for the lower feed intake. On the other hand, HSWI was not associated with any VFA, except for iso-C4 at 7 d. Generally, housing recommendations suggest that weaned calves should remain in their original pens for 7–10 days after weaning before having to adjust to new housing conditions [3]. However, this was not the case for the majority of calves in our study due to space limitations or the management practices applied; most calves (individual or group housed) were transferred to a different barn immediately after weaning. Consequently, HSWI may not be a main factor associated with VFA concentrations and pH post-weaning.

Calves fed low amounts of MR had higher C5 concentration, which mainly indicates higher starter consumption; however, other VFAs related to starter consumption (C3 and C4) were not associated with VOLM within the range fed in the present study and when it was included as a main effect in the models. Low VOLM-fed calves appear to be more affected by weaning based on several rumen parameter compared to those fed higher volumes of MR, but their starter intake is still always higher. High C3 concentration indicates high starter intake, which is also supported by lower pH values. The drop in C3 concentration and the increased pH value at 0 d, depicting a reduced starter intake, were indicative of weaning stress. Propionate concentration rebounded promptly (7 d), but the decreased C2 concentration and C2:C3 ratio (1.18) indicated low forage consumption post-weaning.

Data regarding the less abundant VFAs, that is, C6 and C7, are rather scarce [39]; their role in ruminal function and adaptation must be studied and elucidated before assumptions regarding optimal concentrations can be made.

## 5. Conclusions

A large inter-individual phenotypical variability in calf ruminal parameters during the peri-weaning period was found. Increased AGEW and BW-7 were positively associated with successful rumen adaptation. Feeding calves up to 8 L of MR daily had no direct effects on rumen parameters in this study. Although evidence is not decisive, step weaning appears preferable. Group housing pre-weaning and late forage feeding (after the 5th week, in long form) were the management strategies definitively resulting in a successful ruminal adaptation.

## Figures and Tables

**Table 1:** Management practices applied in the eight farms participating in the study.

		Farms							
		A	B	D	E	F	G	H	I
	No of Calves	7	34	28	34	40	11	51	31
**Age at weaning (±SD)**		68.7 (±14.8)	55.2 (±5.9)	70.5 (±7.7)	80.5 (±7.7)	64.6 (±8.5)	82.6 (±19.9)	64.3 (±6.9)	89.9 (±7.4)
**Milk Replacer**	Amount (L)	5	6	6	8	6	7	6	4
	CP%	22.0	22.0	21.5	22.0	22.0	21.5	22.5	22.5
	Fat%	18.0	18.0	18.0	18.0	18.0	18.0	22.0	22.0
**Starter**	CP%	18.4	19.0	17.0	17.2	17.8	17.2	18.1	20.0
	NDF%	18	20	18	21	19	19	20	20
**Method of weaning**	Abrupt	7	-	28	8	-	-	7	-
	Step-down	-	34	-	26	40	11	44	31
**Forage inclusion pre-weaning**	No	7	-	9	-	-	-	18	3
	Early	-	-	-	34	40	11	18	28
	Late	-	34	19	-	-	-	14	-
**Forage inclusion post-weaning**	No	7	-	-	-	-	-	-	-
	Yes	-	34	28	34	40	11	51	31
**Housing pre-weaning**	Individual	7	34	5	26	40	9	26	-
	Group	-	-	23	8	-	2	25	31
**Housing post-weaning**	Individual	7	30	3	13	25	9	23	4
	Group	-	4	25	21	15	2	28	27

**Table 2:** Descriptive statistics for ruminal volatile fatty acid (VFA) concentration and pH values for the peri-weaning period in Holstein dairy calves. A total of 242, 241 and 239 samples were available at −7 d (7 days pre-weaning), at 0 d (weaning), and 7 d (7 days post-weaning), respectively.

	Descriptive Statistics					
Variable	Time-Points (d)	Mean	SD	Median	95% CI for the Mean	
					Lower Bound	Upper Bound
**Total VFA (mmol/L)**	−7	118.78	43.39	113.79	113.29	124.28
	0	111.11	40.29	106.05	106.00	116.22
	7	107.42	33.39	104.21	103.16	111.67
	Overall	112.46	39.50	107.65	109.57	115.35
**Acetate (mmol/L)**	−7	59.35	19.15	59.56	56.93	61.78
	0	55.83	16.76	54.75	53.70	57.95
	7	54.04	14.94	53.90	52.14	55.94
	Overall	56.42	17.16	55.91	55.16	57.67
**Propionate (mmol/L)**	−7	37.87	20.41	32.83	35.28	40.45
	0	35.45	20.21	29.32	32.89	38.01
	7	33.52	16.88	29.17	31.37	35.67
	Overall	35.62	19.29	30.33	34.21	37.03
**Acetate: Propionate**	−7	1.85	0.75	1.68	1.76	1.95
	0	1.89	0.78	1.79	1.79	1.99
	7	1.96	0.84	1.75	1.85	2.10
	Overall	1.90	0.79	1.75	1.84	1.96
**Butyrate (mmol/L)**	−7	14.37	6.43	9.37	13.56	15.19
	0	13.14	5.89	8.78	12.40	13.89
	+7	13.39	5.88	9.33	12.65	14.14
	Overall	13.64	6.09	9.23	13.19	14.08
**Isobutyrate (mmol/L)**	−7	0.84	0.77	1.36	0.75	0.94
	0	0.86	0.79	1.28	0.76	0.96
	7	0.74	0.67	1.32	0.66	0.83
	Overall	0.81	0.75	1.33	0.76	0.87
**Valerate (mmol/L)**	−7	3.81	2.12	3.21	1.37	1.57
	0	3.43	2.03	2.76	1.27	1.50
	7	3.31	1.95	2.66	1.22	1.45
	Overall	3.52	2.05	2.93	1.34	1.46
**Isovalerate (mmol/L)**	−7	1.47	0.81	1.31	3.54	4.08
	0	1.39	0.91	1.19	3.18	3.69
	7	1.34	0.90	1.11	3.06	3.56
	Overall	1.40	0.87	1.22	3.37	3.67
**Caproate (mmol/L)**	−7	0.87	0.66	0.74	0.78	0.95
	0	0.83	0.61	0.62	0.75	0.90
	7	0.89	0.63	0.70	0.81	0.97
	Overall	0.86	0.63	0.67	0.81	0.91
**Enanthate (mmol/L)**	−7	0.20	0.12	0.18	0.18	0.21
	0	0.19	0.08	0.16	0.17	0.20
	7	0.20	0.09	0.17	0.18	0.21
	Overall	0.19	0.10	0.17	0.19	0.20
**pH**	−7	6.07	0.78	6.10	5.97	6.17
	0	6.32	0.74	6.48	6.22	6.41
	7	6.21	0.79	6.30	6.11	6.32
	Overall	6.20	0.78	6.30	6.14	6.26

CI: confidence interval.

**Table 3:** Estimated marginal means (EMM) of volatile fatty acid (VFA) concentration and pH at 3 time-points (−7 d: 7 days pre-weaning, at 0 d: weaning and 7 d: 7 days post-weaning) from 243 Holstein calves of eight commercial dairy farms.

Estimated Marginal Means					
Time-Points					*p*-Value
Parameter	Unit	−7 d (95% CI)	0 d (95% CI)	7 d (95% CI)	Time
**Total VFA**	mmol/L	117.98 ^a^ (109.12–126.84)	114.43 ^a, b^ (106.84–122.01)	108.74 ^b^ (101.11–116.37)	0.028
**Acetate**	mmol/L	58.77 ^a^ (55.55–61.98)	55.36 ^b^ (52.15–58.57)	54.54 ^b^ (51.31–57.78)	0.013
**Propionate**	mmol/L	-	-	-	0.596
**Acetate: Propionate**		2.30 ^a^ (2.10–2.49)	2.25 ^a^ (2.09–2.41)	1.78 ^b^ (1.65–1.91)	0.001
**Butyrate**	mmol/L	16.27 ^a^ (14.14–18.40)	15.04 ^b^ (12.91–17.17)	10.62 ^b^ (8.72–12.52)	<0.001
**Isobutyrate**	mmol/L	0.77 ^a^ (0.55–1.00)	1.00 ^b^ (0.81–1.19)	0.64 ^a^ (0.45–0.84)	0.001
**Valerate**	mmol/L	3.37 ^a^ (2.93–3.81)	2.96 ^b^ (2.52–3.40)	3.23 ^a, b^ (2.87–3.59)	0.004
**Isovalerate**	mmol/L	1.67 ^a^ (1.47–1.86)	1.64 ^a^ (1.44–1.84)	1.23 ^b^ (1.10–1.38)	0.001
**Caproate**	mmol/L	-	-	-	0.192
**Enanthate**	mmol/L	-	-	-	0.615
**pH**		-	-	-	0.688

^a,b^ Different superscripts within the same row denote significant differences at the 0.05 level.

**Table 4:** Effect of factors examined on the concentration of ruminal volatile fatty acids (VFA) and pH values (factor with a significant effect in bold) determined in 243 Holstein dairy calves of eight commercial dairy farms at 3 different time-points (−7 d: 7 days pre-weaning, at 0 d: weaning, and 7 d: 7 days post-weaning).

*p*-Values											
Parameter	Total VFA	Acetate	Propionate	Acetate: Propionate	Butyrate	Isobutyrate	Valerate	Isovalerate	Caproate	Enanthate	pH
**Factors**											
AGEW	**0.028**	**0.019**	0.071	0.334	**<0.001**	0.178	**0.022**	**0.001**	**0.019**	0.315	0.994
BW-7	**0.003**	**0.001**	**0.017**	0.797	0.402	**<0.001**	**0.007**	**0.001**	0.858	0.401	0.188
VOLM	0.364	0.725	0.211	0.114	0.153	0.678	**<0.001**	0.890	0.195	**<0.001**	0.744
METW	0.333	0.318	0.245	0.354	0.565	0.142	0.382	0.584	0.996	0.421	**0.001**
FPRE	**<0.001**	0.294	**<0.001**	**<0.001**	0.740	0.994	**<0.001**	0.994	**0.001**	**0.020**	**<0.001**
FPOS	0.324	0.174	0.998	0.591	**0.012**	0.254	0.875	0.401	0.964	0.933	0.409
HPRE	**<0.001**	**<0.001**	**<0.001**	**<0.001**	0.444	**0.004**	0.096	0.240	0.641	0.467	**<0.001**
HSWI	0.223	0.517	0.255	0.602	0.386	**0.006**	0.383	0.421	0.462	0.300	0.277
HPOS	0.213	0.839	**0.034**	**<0.001**	0.175	**0.016**	**0.004**	**0.016**	0.155	0.220	0.360

AGEW: Age at weaning; BW-7: BW at −7 d; VOLM: Daily volume of MR; METW: Method of weaning; FPRE: Forage administration pre-weaning; FPOS: Forage inclusion post-weaning; HPRE: Housing pre-weaning; HSWI: Switch housing at weaning; HPOS: Housing post-weaning.

**Table 5:** Estimated marginal means (EMM) of volatile fatty acid (VFA) concentration and pH, of factors having a significant effect at 3 time-points (−7 d: 7 days pre-weaning, at 0 d: weaning, and 7 d: 7 days post-weaning) from 243 Holstein calves of eight commercial dairy farms.

Estimated Marginal Means										
Factors	Level	Total VFA	Acetate	Propionate	Acetate: Propionate	Butyrate	Isobutyrate	Valerate	Isovalerate	pH
**VOLM**	**Low**								4.20 ^a^ (3.57–4.82)	
	**Medium**								3.12 ^b^ (2.80–3.44)	
	**High**								2.25 ^c^ (1.64–2.86)	
**METW**	**Abrupt**									6.70 ^b^ (6.44–6.96)
	**Step-Weaning**									6.04 ^a^ (5.94–6.14)
**FPRE**	**No**	107.83 ^a^ (97.66–118.00)		31.82 ^a^ (26.60–37.03)	2.06 ^a, b^ (1.84–2.28)			2.10 ^a^ (1.55–2.63)		6.23 ^b^ (6.02–6.44)
	**Early**	106.11 ^a^ (97.75–114.48)		30.73 ^a^ (26.36–35.10)	2.22 ^a^ (2.05–2.39)			2.91 ^b^ (2.54–3.27)		6.54 ^a^ (6.37–6.71)
	**Late**	127.20 ^b^ (117.10–137.31)		47.00 ^b^ (41.84–52.16)	1.87 ^b^ (1.71–2.03)			4.57 ^c^ (4.05–5.08)		6.13 ^b^ (5.99–6.26)
**FPOS**	**No**					11.58 ^a^ (10.22–12.94)				
	**Yes**					16.38 ^b^ (13.67–19.09)				
**HPRE**	**Individual**	104.12 ^a^ (96.39–11.86)	52.28 ^a^ (49.35–56.00)	30.61 ^a^ (26.19–35.03)	2.33 ^a^ (2.15–2.51)		0.96 ^a^ (0.78–1.15)			6.73 ^a^ (6.57–6.89)
	**Group**	123.31 ^b^ (114.20–132.41)	60.17 ^b^ (56.58–63.78)	42.42 ^b^ (37.82–47.03)	1.89 ^b^ (1.73–2.05)		0.65 ^b^ (0.45–0.84)			6.01 ^b^ (5.86–6.16)
**HSWI**	**No**						1.04 ^a^ (0.84–1.25)			
	**Yes**						0.57 ^b^ (0.31–0.82)			
**HPOS**	**Individual**			39.35 ^a^ (35.74–42.97)	1.77 ^a^ (1.63–1.90)		0.55 ^a^ (0.30–0.81)	3.59 ^a^ (3.25–3.92)	1.35 ^a^ (1.22–1.47)	
	**Group**			33.67 ^b^ (28.22–39.13)	2.45 ^b^ (2.25–2.66)		1.06 ^b^ (0.80–1.32)	2.79 ^b^ (2.26–3.32)	1.68 ^b^ (1.44–1.92)	

^a–c^ Different superscripts within the same column for each factor denote significant differences at the 0.05 level. VOLM: Daily volume of milk replacer; METW: Method of weaning; FPRE: Forage administration pre-weaning; FPOS: Forage inclusion post-weaning; HPRE: Housing pre-weaning; HSWI: Switch housing at weaning; HPOS: Housing post-weaning. Daily volume of milk replacer (“low” (4–5 L), “medium” (6 L) and “high” (7–8 L)). Forage administration pre-weaning (“no”, “early” (before 1st month of age), and “late” administration (after 1st month of age)).

**Table 6:** Effect of factors as two-way interactions examined on the concentration of ruminal volatile fatty acids (VFA) and pH (factors with a significant effect in bold) values determined in 243 Holstein dairy calves of eight commercial dairy farms at 3 time-points (7 days pre-weaning (−7 d), weaning and 7 days post-weaning).

*p*-Values											
Parameter	Total VFA	Acetate	Propionate	Acetate: Propionate	Butyrate	Isobutyrate	Valerate	Isovalerate	Caproate	Enanthate	pH
**Factors**											
TIME× VOLM	0.138	**0.015**	**0.003**	**0.001**	0.183	**<0.001**	0.648	**0.001**	0.321	0.115	**0.005**
TIME× FPRE	**0.008**	0.372	**0.004**	**<0.001**	0.149	**0.001**	**<0.001**	0.570	0.148	0.412	**0.001**
TIME× HPRE	0.206	0.550	**0.023**	**<0.001**	0.395	**0.025**	**0.043**	**0.057**	0.717	0.215	**0.038**
METW× VOLM	**<0.001**	0.215	**<0.001**	**0.001**	0.441	**0.011**	0.256	0.802	**0.008**	0.176	**0.005**
METW× FPRE	0.646	0.264	0.402	0.060	0.203	0.870	0.284	0.358	0.253	0.342	0.233
VOLM× AGEW	0.105	**0.001**	0.789	0.620	0.104	0.294	0.909	0.171	0.921	**0.004**	0.270
VOLM× BW-7	**0.001**	**0.001**	**0.001**	**0.034**	**0.001**	0.495	**<0.001**	0.129	0.196	**0.003**	**0.001**

TIME: Time-point; VOLM: Daily volume of MR; FPRE: Forage administration pre-weaning; HPRE: Housing pre-weaning; METW: Method of weaning; HPOS: Housing post-weaning; AGEW: Age at weaning; BW-7: BW at −7 d.

## Data Availability

The data presented in this study are available on request from the corresponding author.

## Supplementary Materials

The following supporting information can be downloaded at: https://www.mdpi.com/article/10.3390/ani12070894/s1, Table S1: Estimated marginal means (EMM) of Caproate and Enanthate concentration, of factors having a significant effect at 3 time-points (−7 d: 7 days pre-weaning, at 0 d: weaning and 7 d: 7 days post-weaning) from 243 Holstein calves of 8 commercial dairy farms. Table S2: Estimated marginal means (EMM) showing the variation of total volatile fatty acid (VFA) concentration for all variables as 2-way interactions with significant effect, measured in 243 Holstein dairy calves of 8 commercial dairy farms at 3 time-points [7 days pre-weaning, at weaning (0 d) and 7 days post-weaning], Table S3: Estimated marginal means (EMM) showing the variation of acetate concentration for all variables as 2-way interactions with significant effect, measured in 243 Holstein dairy calves of 8 commercial dairy farms at 3 time-points [7 days pre-weaning, at weaning (0 d) and 7 days post-weaning], Table S4: Estimated marginal means (EMM) showing the variation of Propionate for all variables as 2-way interactions with significant effect, measured in 243 Holstein dairy calves of 8 commercial dairy farms at 3 time-points [7 days pre-weaning, at weaning (0 d) and 7 days post-weaning], Table S5: Estimated marginal means (EMM) showing the variation of acetate: propionate ratio for all variables as 2-way interactions with significant effect, measured in 243 Holstein dairy calves of 8 commercial dairy farms at 3 time-points [7 days pre-weaning, at weaning (0 d) and 7 days post-weaning], Table S6: Estimated marginal means (EMM) showing the variation of isobutyrate concentration for all variables as 2-way interactions with significant effect, measured in 243 Holstein dairy calves of 8 commercial dairy farms at 3 time-points [7 days pre-weaning, at weaning (0 d) and 7 days post-weaning], Table S7: Estimated marginal means (EMM) showing the variation of valerate concentration for all variables as 2-way interactions with significant effect, measured in 243 Holstein dairy calves of 8 commercial dairy farms at 3 time-points [7 days pre-weaning, at weaning (0 d) and 7 days post-weaning], Table S8: Estimated marginal means (EMM) showing the variation of isovalerate concentration for all variables as 2-way interactions with significant effect, measured in 243 Holstein dairy calves of 8 commercial dairy farms at 3 time-points [7 days pre-weaning, at weaning (0 d) and 7 days post-weaning], Table S9: Estimated marginal means (EMM) showing the variation of caproate concentration for all variables as 2-way interactions with significant effect, measured in 243 Holstein dairy calves of 8 commercial dairy farms at 3 time-points [7 days pre-weaning, at weaning (0 d) and 7 days post-weaning], Table S10: Estimated marginal means (EMM) showing the variation of pH values for all variables as 2-way interactions with significant effect, measured in 243 Holstein dairy calves of 8 commercial dairy farms at 3 time-points [7 days pre-weaning, at weaning (0 d) and 7 days post-weaning].
