# Supplementary material for: Phenotypical Variation of Ruminal Volatile Fatty Acids and pH during the Peri-Weaning Period in Holstein Calves and Factors Affecting Them
Source: Animals (Basel). 2022 Mar 31;12(7):894. doi: 10.3390/ani12070894 (PMC8996918; doi:10.3390/ani12070894)
Supplement: Supplementary file 1 [file animals-12-00894-s001.zip › animals-1650271-supplementary/S1.pdf]

Supplementary Table S1. Estimated marginal means (EMM) of Caproate and Enanthate concentration, of factors having a significant effect at 3 time-points (-7d : 7 days pre-weaning, at 0d: weaning and 7d: 7 days post-weaning) from 243 Holstein calves of 8 commercial dairy farms.

		**Estimated marginal means**	
**Factors**	**Level**	**Caproate**	**Enanthate**
**VOLM**	**Low**		0.20 (0.16-2.43)
	**Medium**		0.19 (0.17-0.20)
	**High**		0.15 (0.11-0.18)
**FPRE**	**No**	0.63[a] (0.48-0.79)	0.16[a] (0.133-0.19)
	**Early**	0.90[b] (0.76-1.03)	0.17[a, b] (0.16-0.19)
	**Late**	0.87[a, b] (0.74-1.00)	0.20[b] (0.17-0.23)

a-b Different superscripts within the same column for each factor denote significant differences at the 0.05 level.
VOLM: Daily volume of MR; FPRE: Forage administration pre-weaning;
Daily volume of Milk Replacer [“low” (4-5 L), “medium” (6 L) and “high” (7-8 L)].
Forage administration pre-weaning [“no”, “early” (before 1st month of age) and “late” administration (after 1st month of age)].
