# Supplementary material for: Phenotypical Variation of Ruminal Volatile Fatty Acids and pH during the Peri-Weaning Period in Holstein Calves and Factors Affecting Them
Source: Animals (Basel). 2022 Mar 31;12(7):894. doi: 10.3390/ani12070894 (PMC8996918; doi:10.3390/ani12070894)
Supplement: Supplementary file 1 [file animals-12-00894-s001.zip › animals-1650271-supplementary/S10.pdf]

**Supplementary Table S10.** Estimated marginal means (EMM) showing the variation of pH values for all variables as 2-way interactions with significant effect, measured in 243 Holstein dairy calves of 8 commercial dairy farms at 3 time-points [7 days pre-weaning, at weaning (0d) and 7 days post-weaning].

| | **pH** | | | | | |
|---|---|---|---|---|---|---|
| | Daily Volume of Milk Replacer | | | | | |
| | **Low** | | **Medium** | | **High** | |
| **Time-points** | **EMM (95% CI)** | **SE** | **EMM (95% CI)** | **SE** | **EMM (95% CI)** | **SE** |
| -7d | $6.36^{ab. A}$ (5.93-6.78) | 0.22 | $6.36^{a. A}$ (6.17-6.55) | 0.10 | $6.48^{a. A}$ (6.15-6.81) | 0.17 |
| 0d | $6.67^{a. A}$ (6.36-6.98) | 0.16 | $6.30^{a. A}$ (6.18-6.42) | 0.06 | $6.39^{a. A}$ (6.12-6.65) | 0.14 |
| 7d | $6.21^{b. A}$ (5.81-6.61) | 0.20 | $6.30^{a. A}$ (6.17-6.42) | 0.06 | $6.39^{a. A}$ (6.15-6.64) | 0.13 |
| | Forage administration pre-weaning | | | | | |
| | **No** | | **Early** | | **Late** | |
| **Time-points** | **EMM (95% CI)** | **SE** | **EMM (95% CI)** | **SE** | **EMM (95% CI)** | **SE** |
| -7d | $6.41^{a. AB}$ (6.11-6.70) | 0.15 | $6.46^{a. A}$ (6.25-6.66) | 0.10 | $6.10^{a. B}$ (5.87-6.33) | 0.12 |
| 0d | $6.20^{a. A}$ (5.96-6.44) | 0.12 | $6.73^{b. B}$ (6.54-6.91) | 0.09 | $6.06^{a. A}$ (5.89-6.23) | 0.09 |
| 7d | $6.09^{a. A}$ (5.78-6.39) | 0.16 | $6.43^{a. A}$ (6.25-6.62) | 0.09 | $6.22^{a. A}$ (6.05-6.39) | 0.09 |
| | Housing pre-weaning | | | | | |
| | **Individual** | | | | **Group** | |
| **Time-points** | **EMM (95% CI)** | **SE** | | | **EMM (95% CI)** | **SE** |
| -7d | $6.63^{a. A}$ (6.42-6.83) | 0.10 | | | $6.13^{ab. B}$ (5.92-6.34) | 0.11 |

| | | | | | | |
|---|---|---|---|---|---|---|
| 0d | $6.83^{a. A}$ (6.65-7.00) | 0.09 | | | $6.05^{a. B}$ (5.86-6.24) | 0.10 |
| 7d | $6.74^{a. A}$ (6.53-6.94) | 0.10 | | | $5.83^{b. B}$ (5.64-6.03) | 0.10 |

| | Daily Volume of Milk Replacer | | | | | |
|---|---|---|---|---|---|---|
| | **Low** | | **Medium** | | **High** | |
| **Method of weaning** | **EMM (95% CI)** | **SE** | **EMM (95% CI)** | **SE** | **EMM (95% CI)** | **SE** |
| Step down | $6.00^{a, A}$ (5.78-6.21) | 0.11 | $5.93^{a, A}$ (5.81-6.04) | 0.06 | $6.44^{a, B}$ (6.28-6.59) | 0.08 |
| Abrupt | $6.83^{b, A}$ (6.20-7.46) | 0.32 | $6.71^{b, A}$ (6.47-6.95) | 0.12 | $6.40^{a, A}$ (5.95-6.85) | 0.23 |

SE: Standard error

a-b Different superscripts within the same column denote significant differences at the 0.05 level.

A-B Different superscripts within the same row denote significant differences at the 0.05 level.

Daily volume of Milk Replacer [“low” (4-5 L), “medium” (6 L) and “high” (7-8 L)].

Forage administration pre-weaning [“no”, “early” (before 1st month of age) and “late” administration (after 1st month of age)].
