# Supplementary material for: Phenotypical Variation of Ruminal Volatile Fatty Acids and pH during the Peri-Weaning Period in Holstein Calves and Factors Affecting Them
Source: Animals (Basel). 2022 Mar 31;12(7):894. doi: 10.3390/ani12070894 (PMC8996918; doi:10.3390/ani12070894)
Supplement: Supplementary file 1 [file animals-12-00894-s001.zip › animals-1650271-supplementary/S2.pdf]

Supplementary Table S2. Estimated marginal means (EMM) showing the variation of total volatile fatty acid (VFA) concentration for all variables as 2-way interactions with significant effect, measured in 243 Holstein dairy calves of 8 commercial dairy farms at 3 time-points [7 days pre-weaning, at weaning (0d) and 7 days post-weaning].

Total VFA						
	Forage administration pre-weaning					
	No		Early		Late	
Time-points	EMM (95% CI)	SE	EMM (95% CI)	SE	EMM (95% CI)	SE
-7d	$109.74^{a, A}$ (96.38-123.11)	6.80	$110.41^{a, A}$ (100.81-120.00)	4.89	$133.78^{a, B}$ (121.81-145.74)	6.09
0d	$107.35^{a, A}$ (94.61-120.09)	6.49	$102.78^{a, A}$ (93.70-111.85)	4.62	$133.15^{ab, B}$ (121.72-144.58)	5.82
7d	$106.39^{a, A}$ (93.55-119.24)	6.54	$105.15^{a, A}$ (96.06-114.24)	4.63	$114.68^{b, A}$ (103.20-126.16)	5.84
	Daily volume of MR					
Method of weaning	Low		Medium		High	
	EMM (95% CI)	SE	EMM (95% CI)	SE	EMM (95% CI)	SE
Step down	$131.51^{a, A}$ (119.91-143.11)	5.89	$116.55^{a, AB}$ (110.39-122.70)	3.13	$102.44^{a, B}$ (90.78-114.11)	5.93
Abrupt	$119.21^{a, A}$ (93.93-144.48)	12.87	$98.43^{b, A}$ (87.59-109.28)	5.52	$114.14^{a, A}$ (92.28-135.99)	11.13

SE: Standard error

a-b Different superscripts within the same column denote significant differences at the 0.05 level.

A-B Different superscripts within the same row denote significant differences at the 0.05 level.

Forage administration pre-weaning [“no”, “early” (before 1st month of age) and “late” administration (after 1st month of age)].

Daily volume of MR [“low” (4-5 L), “medium” (6 L) and “high” (7-8 L)].
