# Supplementary material for: Phenotypical Variation of Ruminal Volatile Fatty Acids and pH during the Peri-Weaning Period in Holstein Calves and Factors Affecting Them
Source: Animals (Basel). 2022 Mar 31;12(7):894. doi: 10.3390/ani12070894 (PMC8996918; doi:10.3390/ani12070894)
Supplement: Supplementary file 1 [file animals-12-00894-s001.zip › animals-1650271-supplementary/S3.pdf]

Supplementary Table S3. Estimated marginal means (EMM) showing the variation of acetate concentration for all variables as 2-way interactions with significant effect, measured in 243 Holstein dairy calves of 8 commercial dairy farms at 3 time-points [7 days pre-weaning, at weaning (0d) and 7 days post-weaning].

	**Acetate**					
	Daily Volume of Milk Replacer					
	**Low**		**Medium**		**High**	
**Time-points**	**EMM (95% CI)**	**SE**	**EMM (95% CI)**	**SE**	**EMM (95% CI)**	**SE**
-7d	$59.91^{a, A}$ (52.80-67.02)	3.62	$61.32^{a, A}$ (58.34-64.30)	1.52	$55.07^{a, A}$ (49.31-60.83)	2.93
0d	$52.56^{b, A}$ (45.47-59.64)	3.60	$57.50^{b, A}$ (54.52-60.48)	1.52	$56.02^{a, A}$ (50.26-61.79)	2.93
7d	$50.33^{b, A}$ (43.22-57.44)	3.62	$54.47^{b, A}$ (51.47-57.46)	1.52	$58.83^{a, A}$ (52.97-64.69)	2.98

SE: Standard error

a-b Different superscripts within the same column denote significant differences at the 0.05 level.

A-B Different superscripts within the same row denote significant differences at the 0.05 level.

Daily volume of Milk Replacer [“low” (4-5 L), “medium” (6 L) and “high” (7-8 L)].
