# Supplementary material for: Phenotypical Variation of Ruminal Volatile Fatty Acids and pH during the Peri-Weaning Period in Holstein Calves and Factors Affecting Them
Source: Animals (Basel). 2022 Mar 31;12(7):894. doi: 10.3390/ani12070894 (PMC8996918; doi:10.3390/ani12070894)
Supplement: Supplementary file 1 [file animals-12-00894-s001.zip › animals-1650271-supplementary/S4.pdf]

Supplementary Table S4. Estimated marginal means (EMM) showing the variation of Propionate for all variables as 2-way interactions with significant effect, measured in 243 Holstein dairy calves of 8 commercial dairy farms at 3 time-points [7 days pre-weaning, at weaning (0d) and 7 days post-weaning].

**Propionate**						
	Daily Volume of Milk Replacer					
	**Low**		**Medium**		**High**	
**Time-points**	**EMM (95% CI)**	**SE**	**EMM (95% CI)**	**SE**	**EMM (95% CI)**	**SE**
-7d	$50.44^{a, A}$ (40.58-60.30)	5.02	$27.19^{a, B}$ (21.95-32.42)	2.66	$29.32^{a, B}$ (19.82-38.82)	4.84
0d	$41.86^{b, A}$ (33.72-50.00)	4.14	$29.98^{a, B}$ (25.93-34.02)	2.06	$35.56^{a, AB}$ (27.45-43.67)	4.13
7d	$50.42^{a, A}$ (42.74-58.10)	3.91	$28.98^{a, B}$ (25.68-32.27)	1.68	$34.90^{a, B}$ (27.29-42.51)	3.88
	Forage administration pre-weaning					
	**No**		**Early**		**Late**	
**Time-points**	**EMM (95% CI)**	**SE**	**EMM (95% CI)**	**SE**	**EMM (95% CI)**	**SE**
-7d	$29.15^{a, A}$ (21.87-36.42)	3.70	$30.33^{ab, A}$ (24.88-35.79)	2.78	$47.47^{a, B}$ (40.39-54.54)	3.60
0d	$30.70^{a, A}$ (23.86-37.54)	4.48	$27.68^{a, A}$ (22.56-32.79)	2.60	$49.02^{a, B}$ (42.65-55.39)	3.24
7d	$35.60^{s, A}$ (29.32-41.88)	3.20	$34.18^{b, A}$ (29.65-38.71)	2.31	$44.51^{a, B}$ (38.71-50.31)	2.95
	Housing pre-weaning					
	**Individual**				**Group**	
**Time-points**	**EMM (95% CI)**	**SE**			**EMM (95% CI)**	**SE**
-7d	$32.72^{a, A}$ (27.13-38.32)	2.85			$38.58^{a, B}$ (32.10-45.05)	3.30
0d	$29.39^{a, A}$	2.56			$42.21^{a, B}$	3.15

	(24.37-34.41)				(36.03-48.39)	
7d	29.71$^{a, A}$<br>(25.21-34.22)	2.29			46.48$^{a, B}$<br>(40.63-52.33)	3.00

	Daily Volume of Milk Replacer					
	**Low**		**Medium**		**High**	
**Method of weaning**	**EMM (95% CI)**	**SE**	**EMM (95% CI)**	**SE**	**EMM (95% CI)**	**SE**
Step down	43.89$^{a, A}$<br>(38.04-49.74)	2.97	36.47$^{a, AB}$<br>(32.81-40.13)	1.86	31.74$^{a, B}$<br>(25.60-37.88)	3.12
Abrupt	51.25$^{a, A}$<br>(38.55-63.95)	6.47	21.00$^{b, B}$<br>(14.80-27.11)	3.13	34.78$^{a, A}$<br>(23.49-46.07)	5.75

SE: Standard error

a-b Different superscripts within the same column denote significant differences at the 0.05 level.

A-B Different superscripts within the same row denote significant differences at the 0.05 level.

Daily volume of Milk Replacer [“low” (4-5 L), “medium” (6 L) and “high” (7-8 L)].

Forage administration pre-weaning [“no”, “early” (before 1st month of age) and “late” administration (after 1st month of age)].
