# Supplementary material for: Phenotypical Variation of Ruminal Volatile Fatty Acids and pH during the Peri-Weaning Period in Holstein Calves and Factors Affecting Them
Source: Animals (Basel). 2022 Mar 31;12(7):894. doi: 10.3390/ani12070894 (PMC8996918; doi:10.3390/ani12070894)
Supplement: Supplementary file 1 [file animals-12-00894-s001.zip › animals-1650271-supplementary/S5.pdf]

Supplementary Table S5. Estimated marginal means (EMM) showing the variation of acetate: propionate ratio for all variables as 2-way interactions with significant effect, measured in 243 Holstein dairy calves of 8 commercial dairy farms at 3 time-points [7 days pre-weaning, at weaning (0d) and 7 days post-weaning].

	**Acetate: Propionate**					
	Daily Volume of Milk Replacer					
	**Low**		**Medium**		**High**	
**Time-points**	**EMM (95% CI)**	**SE**	**EMM (95% CI)**	**SE**	**EMM (95% CI)**	**SE**
-7d	$1.92^{a, A}$ (1.52-2.32)	0.20	$2.52^{a, B}$ (2.31-2.73)	0.11	$2.38^{a, AB}$ (2.05-2.72)	0.17
0d	$1.99^{a, A}$ (1.67-2.31)	0.16	$2.42^{a, B}$ (2.26-2.58)	0.08	$2.26^{ab, AB}$ (2.00-2.52)	0.13
7d	$1.18^{b, A}$ (0.87-1.48)	0.15	$2.12^{b, B}$ (1.99-2.25)	0.07	$1.97^{b, B}$ (1.72-2.22)	0.13
	Forage administration pre-weaning					
	**No**		**Early**		**Late**	
**Time-points**	**EMM (95% CI)**	**SE**	**EMM (95% CI)**	**SE**	**EMM (95% CI)**	**SE**
-7d	$2.29^{a, AB}$ (1.98-2.59)	0.15	$2.41^{a, A}$ (2.20-2.63)	0.11	$1.96^{a, B}$ (1.71-2.22)	0.13
0d	$2.19^{a, A}$ (1.92-2.46)	0.14	$2.44^{a, A}$ (2.24-2.64)	0.10	$1.82^{a, B}$ (1.62-2.02)	0.10
7d	$1.70^{b, A}$ (1.44-1.95)	0.13	$1.82^{b, A}$ (1.65-2.00)	0.09	$1.82^{a, A}$ (1.65-2.00)	0.09
	Housing pre-weaning					
	**Individual**				**Group**	
**Time-points**	**EMM (95% CI)**	**SE**			**EMM (95% CI)**	**SE**

-7d	$2.38^{ab, A}$ (2.16-2.60)	0.11			$2.21^{a, A}$ (1.98-2.44)	0.12
0d	$2.43^{a, A}$ (2.23-2.63)	0.10			$2.07^{a, B}$ (1.86-2.29)	0.11
7d	$2.17^{b, A}$ (1.99-2.35)	0.09			$1.39^{b, B}$ (1.18-1.60)	0.11

	Daily Volume of Milk Replacer					
	**Low**		**Medium**		**High**	
**Method of weaning**	**EMM (95% CI)**	**SE**	**EMM (95% CI)**	**SE**	**EMM (95% CI)**	**SE**
Step down	$1.67^{a, A}$ (1.43-1.92)	0.12	$2.11^{a, B}$ (1.96-2.25)	0.07	$2.36^{a, C}$ (2.17-2.55)	0.10
Abrupt	$1.72^{a, A}$ (1.19-2.24)	0.27	$2.60^{b, B}$ (2.35-2.85)	0.13	$2.05^{a, A}$ (1.61-2.48)	0.22

SE: Standard error

a-c Different superscripts within the same column denote significant differences at the 0.05 level.

A-C Different superscripts within the same row denote significant differences at the 0.05 level.

Daily volume of Milk Replacer [“low” (4-5 L), “medium” (6 L) and “high” (7-8 L)].

Forage administration pre-weaning [“no”, “early” (before 1st month of age) and “late” administration (after 1st month of age)].
