# Supplementary material for: Phenotypical Variation of Ruminal Volatile Fatty Acids and pH during the Peri-Weaning Period in Holstein Calves and Factors Affecting Them
Source: Animals (Basel). 2022 Mar 31;12(7):894. doi: 10.3390/ani12070894 (PMC8996918; doi:10.3390/ani12070894)
Supplement: Supplementary file 1 [file animals-12-00894-s001.zip › animals-1650271-supplementary/S6.pdf]

Supplementary Table S6. Estimated marginal means (EMM) showing the variation of isobutyrate concentration for all variables as 2-way interactions with significant effect, measured in 243 Holstein dairy calves of 8 commercial dairy farms at 3 time-points [7 days pre-weaning, at weaning (0d) and 7 days post-weaning].

**Isobutyrate**						
	Daily Volume of Milk Replacer					
	**Low**		**Medium**		**High**	
**Time-points**	**EMM (95% CI)**	**SE**	**EMM (95% CI)**	**SE**	**EMM (95% CI)**	**SE**
-7d	$0.61^{a,\ A}$ (0.23-1.0)	0.19	$0.88^{a,\ A}$ (0.66-1.10)	0.11	$0.82^{a,\ A}$ (0.45-1.19)	0.19
0d	$1.28^{b,\ A}$ (0.97-1.60)	0.16	$0.67^{b,\ B}$ (0.50-0.83)	0.09	$1.10^{a,\ AB}$ (0.74-1.37)	0.16
7d	$0.59^{a,\ AB}$ (0.25-0.92)	0.17	$0.46^{b,\ A}$ (0.30-0.61)	0.08	$0.88^{a,\ B}$ (0.57-1.20)	0.16
	Forage administration pre-weaning					
	**No**		**Early**		**Late**	
**Time-points**	**EMM (95% CI)**	**SE**	**EMM (95% CI)**	**SE**	**EMM (95% CI)**	**SE**
-7d	$0.80^{a,\ AB}$ (0.50-1.10)	0.15	$0.95^{a,\ A}$ (0.74-1.16)	0.11	$0.57^{a,\ B}$ (0.27-0.87)	0.15
0d	$0.93^{a,\ A}$ (0.65-1.21)	0.14	$0.97^{a,\ A}$ (0.77-1.17)	0.10	$1.10^{b,\ A}$ (0.83-1.37)	0.14
7d	$0.59^{s,\ A}$ (0.33-0.86)	0.14	$0.52^{b,\ A}$ (0.29-0.75)	0.12	$0.82^{ab,\ A}$ (0.54-1.10)	0.14
	Housing pre-weaning					
	**Individual**				**Group**	
**Time-points**	**EMM (95% CI)**	**SE**			**EMM (95% CI)**	**SE**
-7d	$0.81^{a,\ A}$ (0.58-1.04)	0.12			$0.74^{ab,\ A}$ (0.48-1.00)	0.13

0d	1.13$^{b, A}$ (0.92-1.33)	0.11			0.88$^{a, A}$ (0.63-1.13)	0.13
7d	0.96$^{ab, A}$ (0.77-1.14)	0.10			0.33$^{b, B}$ (-0.01-0.67)	0.17

	Daily Volume of Milk Replacer					
	**Low**		**Medium**		**High**	
**Method of weaning**	**EMM (95% CI)**	**SE**	**EMM (95% CI)**	**SE**	**EMM (95% CI)**	**SE**
Step down	1.17$^{a, A}$ (0.95-1.40)	0.11	0.54$^{a, B}$ (0.39-0.69)	0.8	0.96$^{a, A}$ (25.60-37.88)	0.13
Abrupt	0.49$^{b, A}$ (-0.01-0.98)	0.25	0.80$^{a, A}$ (0.54-1.05)	0.13	0.88$^{a, A}$ (0.45-1.31)	0.22

SE: Standard error

a-b Different superscripts within the same column denote significant differences at the 0.05 level.

A-B Different superscripts within the same row denote significant differences at the 0.05 level.

Daily volume of Milk Replacer [“low” (4-5 L), “medium” (6 L) and “high” (7-8 L)].

Forage administration pre-weaning [“no”, “early” (before 1st month of age) and “late” administration (after 1st month of age)].
