# Supplementary material for: Phenotypical Variation of Ruminal Volatile Fatty Acids and pH during the Peri-Weaning Period in Holstein Calves and Factors Affecting Them
Source: Animals (Basel). 2022 Mar 31;12(7):894. doi: 10.3390/ani12070894 (PMC8996918; doi:10.3390/ani12070894)
Supplement: Supplementary file 1 [file animals-12-00894-s001.zip › animals-1650271-supplementary/S7.pdf]

Supplementary Table S7. Estimated marginal means (EMM) showing the variation of valerate concentration for all variables as 2-way interactions with significant effect, measured in 243 Holstein dairy calves of 8 commercial dairy farms at 3 time-points [7 days pre-weaning, at weaning (0d) and 7 days post-weaning].

**Valerate**						
	Forage administration pre-weaning					
	**No**		**Early**		**Late**	
**Time-points**	**EMM (95% CI)**	**SE**	**EMM (95% CI)**	**SE**	**EMM (95% CI)**	**SE**
-7d	2.22$^{a. A}$ (1.56-2.89)	0.34	2.87$^{ab. A}$ (2.40-3.33)	0.24	5.02$^{a. B}$ (4.40-5.63)	0.31
0d	1.79$^{a. A}$ (1.13-2.46)	0.33	2.54$^{a. A}$ (2.07-3.00)	0.24	4.56$^{ab. B}$ (3.95-5.17)	0.31
7d	2.26$^{a. A}$ (1.64-2.87)	0.31	3.31$^{b. B}$ (2.92-3.71)	0.20	4.12$^{b. C}$ (3.56-4.68)	0.28

	Housing pre-weaning			
	**Individual**		**Group**	
**Time-points**	**EMM (95% CI)**	**SE**	**EMM (95% CI)**	**SE**
-7d	3.23$^{a, A}$ (2.77-3.69)	0.23	3.51$^{a, A}$ (2.94-4.07)	0.29
0d	3.92$^{a, A}$ (2.46-3.38)	0.23	3.00$^{b, A}$ (2.44-3.57)	0.29
7d	2.82$^{a, A}$ (2.43-3.22)	0.20	3.64$^{ab, B}$ (3.09-4.19)	0.28

SE: Standard error

a-c Different superscripts within the same column denote significant differences at the 0.05 level.

A-C Different superscripts within the same row denote significant differences at the 0.05 level.

Forage administration pre-weaning [“no”, “early” (before 1st month of age) and “late” administration (after 1st month of age)].
