# Supplementary material for: Phenotypical Variation of Ruminal Volatile Fatty Acids and pH during the Peri-Weaning Period in Holstein Calves and Factors Affecting Them
Source: Animals (Basel). 2022 Mar 31;12(7):894. doi: 10.3390/ani12070894 (PMC8996918; doi:10.3390/ani12070894)
Supplement: Supplementary file 1 [file animals-12-00894-s001.zip › animals-1650271-supplementary/S8.pdf]

Supplementary Table S8. Estimated marginal means (EMM) showing the variation of isovalerate concentration for all variables as 2-way interactions with significant effect, measured in 243 Holstein dairy calves of 8 commercial dairy farms at 3 time-points [7 days pre-weaning, at weaning (0d) and 7 days post-weaning].

**Isovalerate**						
	Daily Volume of Milk Replacer					
	**Low**		**Medium**		**High**	
**Time-points**	**EMM (95% CI)**	**SE**	**EMM (95% CI)**	**SE**	**EMM (95% CI)**	**SE**
-7d	$1.79^{a, A}$ (1.48-2.11)	0.16	$1.66^{a, A}$ (1.47-1.86)	0.10	$1.54^{a, A}$ (1.22-1.86)	0.16
0d	$2.01^{a, A}$ (1.70-2.33)	0.16	$1.45^{b, B}$ (1.25-1.65)	0.10	$1.46^{a, B}$ (1.14-1.77)	0.16
7d	$1.22^{b, A}$ (0.93-1.50)	0.15	$1.35^{b, A}$ (1.20-1.50)	0.08	$1.12^{a, A}$ (0.83-1.41)	0.15
	Housing pre-weaning					
	Individual				Group	
Time-points	EMM (95% CI)	SE			EMM (95% CI)	SE
-7d	$1.57^{ab, A}$ (1.36-1.78)	0.11			$1.57^{a, A}$ (1.34-1.85)	0.13
0d	$1.68^{a, A}$ (1.47-1.89)	0.11			$1.60^{a, A}$ (1.34-1.85)	0.13
7d	$1.36^{b, A}$ (1.19-1.53)	0.09			$1.10^{b, A}$ (3.09-4.19)	0.13

SE: Standard error

a-b Different superscripts within the same column denote significant differences at the 0.05 level.

A-B Different superscripts within the same row denote significant differences at the 0.05 level.

Daily volume of Milk Replacer [“low” (4-5 L), “medium” (6 L) and “high” (7-8 L)].
