# Supplementary material for: Phenotypical Variation of Ruminal Volatile Fatty Acids and pH during the Peri-Weaning Period in Holstein Calves and Factors Affecting Them
Source: Animals (Basel). 2022 Mar 31;12(7):894. doi: 10.3390/ani12070894 (PMC8996918; doi:10.3390/ani12070894)
Supplement: Supplementary file 1 [file animals-12-00894-s001.zip › animals-1650271-supplementary/S9.pdf]

Supplementary Table S9. Estimated marginal means (EMM) showing the variation of caproate concentration for all variables as 2-way interactions with significant effect, measured in 243 Holstein dairy calves of 8 commercial dairy farms at 3 time-points [7 days pre-weaning, at weaning (0d) and 7 days post-weaning].

Caproate						
	Daily volume of Milk Replacer					
	**Low**		**Medium**		**High**	
**Method of weaning**	**EMM (95% CI)**	**SE**	**EMM (95% CI)**	**SE**	**EMM (95% CI)**	**SE**
Step down	$0.94^{a, A}$ (0.75-1.13)	0.10	$0.93^{a, A}$ (0.83-1.02)	0.05	$0.45^{a, B}$ (0.29-0.60)	0.08
Abrupt	$0.70^{a, A}$ (0.35-1.04)	0.18	$0.77^{a, A}$ (0.62-0.92)	0.08	0.86^{b} (0.53-1.18)	0.17

SE: Standard error

a-b Different superscripts within the same column denote significant differences at the 0.05 level.

A-B Different superscripts within the same row denote significant differences at the 0.05 level.

Daily volume of Milk Replacer [“low” (4-5 L), “medium” (6 L) and “high” (7-8 L)].
